# Knowledge, attitudes, and practices of caregivers with children diagnosed with epilepsy attending a pediatric outpatient clinic: a descriptive, cross-sectional, questionnaire-based study in Addis Ababa, Ethiopia

**DOI:** 10.1186/s12883-024-03766-1

**Published:** 2024-07-22

**Authors:** Absalat Serawit Negussie, Mansour Fayz Dehan, Samuel Ayalew Mekonnen, Tesfaye Getaneh Zelleke

**Affiliations:** 1 Yekatit 12 Hospital Medical College, Addis Ababa, Ethiopia; 2 School of Medicine and Health Sciences, George Washington University, Washington, DC USA; 3 Children’s National Medical Center, Washington, DC USA

**Keywords:** Epilepsy, Knowledge, Attitude, Practice, Children, Caregivers, Ethiopia, Questionnaire

## Abstract

**Background:**

Caregivers’ knowledge and attitudes influence help-seeking behavior and treatment decisions of patients with epilepsy, which in turn significantly impacts epilepsy care. In Ethiopia, epilepsy is often misunderstood, associated with misconceptions and accompanied by persistent negative attitudes. The objective of this study is to assess the knowledge, attitude, and practice of caregivers of children with epilepsy.

**Methods:**

We conducted a hospital-based survey at the Yekatit 12 Hospital Pediatric Neurology Clinic, Addis Ababa, Ethiopia, between May and July 2022. We invited caregivers of children with epilepsy taking one or more daily anti-seizure medications to participate. Caregivers were invited to complete a structured questionnaire with guidance from a trained nurse to estimate knowledge and attitudes towards epilepsy and its treatment. Knowledge and attitudes were categorized as “good” and “favorable” (correct answers to ≥ 50% of questions) or “bad” and “unfavorable” (< 50% correct answers), respectively. Attitudes towards standard care versus non-standard (e.g., spiritual) care were also estimated.

**Results:**

A total of 120 caregivers completed the questionnaire. Many caregivers were familiar with the term ‘epilepsy’, with more than half (51.7%) having heard or read about it previously. The reported causes of epilepsy varied, with birth injury being the most common cause (44 out of 120 caregivers). Notably, there was association between the caregiver’s gender and their knowledge score, with a p-value = 0.05. Caregivers exposed to information about epilepsy through hearing or reading demonstrated significantly higher levels of knowledge, with a p-value < 0.001. Additionally, knowing someone with epilepsy other than the index child was significantly associated with higher knowledge scores (p-value < 0.001). The study also revealed negative attitudes toward epilepsy: for example, 56.7% of surveyed caregivers believed it is unlikely that a child with epilepsy has normal cognitive abilities and 39.1% believed they should never be allowed to attend regular school. Additionally, a high proportion of caregivers (70%) sought alternative treatments (e.g., spiritual help) alongside standard medical care.

**Conclusions:**

A significant knowledge gap was identified among caregivers, revealing prevalent misconceptions and negative attitudes. Improving epilepsy awareness, attitudes, and practices among caregivers will potentially contribute to overall improved quality of life for children with epilepsy.

**Supplementary Information:**

The online version contains supplementary material available at 10.1186/s12883-024-03766-1.

## Background

Epilepsy is one of the most common neurologic disorders affecting approximately 1% of the global population and accounting for 0.5% of the overall burden of disease. Approximately four-fifths of people with epilepsy live in low- or middle-income countries [1]. In children and adolescents, the prevalence of epilepsy is 4–6 per 1000 people, with approximately 80% of epilepsy diagnoses occurring before the age of 18 years and a peak incidence between ages 1–4 years old [1–3]. The pooled incidence rate of epilepsy is 61.4 per 100,000 person-years, and is greater in low-/middle-income countries compared to high-income countries [3]. This higher incidence in developing countries is related to higher risk of acute and chronic brain infections, as well as pre- and postnatal obstetric complications leading to brain damage [2]. In Ethiopia, the prevalence of epilepsy is 5.2 per 1000 people [4].

The treatment gap for epilepsy is a significant issue, particularly in developing countries where epilepsy is most common [4]. Around 90% of people with epilepsy (PWE) do not receive appropriate treatment [5]. Several factors contribute to this, including a shortage of trained healthcare professionals, high treatment costs, limited availability of medications, and disparities in access to care between urban and rural areas [4, 5]. Additionally, the attitudes of patients, families, and the broader community plays a role in hindering access to care [4, 5]. Caregivers’ knowledge, attitudes, and practices are crucial determinants of epilepsy care because they influence the help-seeking behavior of individuals with epilepsy and their families, as well as the goals and decisions of policy makers. Further, the level of knowledge about epilepsy significantly influences individuals’ engagement with care, thus it is an important determinant of health outcomes in epilepsy [4–7].

In Ethiopia, epilepsy continues to be widely misunderstood and associated with misconceptions, both of which contribute to prominent stigmatization of epilepsy [8–12]. Despite some changes over time, negative attitudes toward epilepsy persist [10, 11]. This knowledge gap impacts care, as a person or caregiver misattributing epilepsy to “evil spirits” may opt to seek alternative treatments instead of utilizing standard healthcare services [7]. Previous studies have investigated the Ethiopian public’s knowledge of epilepsy and the prevalence of stigma toward people with epilepsy [3, 8–13] However, there are limited data concerning the knowledge and attitudes of individuals with epilepsy and caregivers of children with epilepsy (CWE). As the gap in knowledge negatively impacts care, addressing this gap offers an opportunity to improve epilepsy outcomes with community-level interventions. This study aimed to identify specific knowledge, attitudes, practices among caregivers for children with epilepsy.

## Materials and methods

### Study design and study setting

A hospital-based, cross-sectional questionnaire study was carried out at the Pediatric Neurology Outpatient Clinic of Yekatit 12 Hospital Medical College in Addis Ababa, Ethiopia, between May 1 and July 30, 2022. The study obtained ethical clearance from both the Yekatit 12 Hospital Medical College and the Children’s National Medical Center institutional review boards (Ref.no STUDY00000115). Caregivers participating in the study were provided with information on the study’s objectives and the data that would be collected. Written informed consent was obtained from each participant.

### Inclusion and exclusion criteria

Caregivers of CWE attending the clinic with their child were invited to participate in the study.

Inclusion criteria:

1. Caregivers of children aged up to 14 years.
2. A diagnosis of epilepsy made by a physician.
3. Caregivers of children currently prescribed one or more daily anti-seizure medications as part of standard treatment (e.g. Phenytoin, Phenobarbital and Sodium valproate).
4. Caregivers of children with neurodevelopmental disorders, e.g., autism, attention-deficit/hyperactivity disorder and intellectual disability, were not excluded.

Exclusion criteria:

1. Caregivers of children with only provoked seizures (seizures only seen in setting of illness or fever such as febrile seizures are excluded).
2. Caregivers of children not currently prescribed and not taking anti-seizure medications.

### Survey questionnaire development

In our study, we conducted an extensive literature review to develop a structured questionnaire, drawing insights from previous research on knowledge and attitudes towards epilepsy in Ethiopia [3, 9, 13, 14]. Based on these insights, we translated the questionnaire from English to Amharic using two professional translators. To ensure the accuracy of the translation, we employed a back-translation method, converting the Amharic version back to English. This iterative process helped identify and resolve any discrepancies, ensuring that the translated questionnaire accurately captured the original content.

Following the translation, we convened an expert committee with diverse backgrounds, including individuals holding M.D. and M.P.H. degrees, as well as pediatricians and child neurologists. This committee was responsible for reviewing and refining the translated questionnaire, resulting in a pre-final version. To test the pre-final version, we conducted a pilot study with 50 caregivers of children with epilepsy. During this phase, caregivers were asked to provide detailed feedback on their understanding and interpretation of each questionnaire item. This feedback was crucial for identifying ambiguities and areas for improvement. Based on the caregivers’ input, we made necessary adjustments to enhance clarity and relevance.

The revised questionnaire was then subjected to validation by the investigators. This validation process involved ensuring that the questionnaire was comprehensive, culturally appropriate, and capable of accurately capturing the intended data. Upon completion of this rigorous modification and validation process, the final version of the questionnaire was administered to the caregivers, ensuring that it was a reliable and effective tool for our study [15].

The questionnaire was then made available to participants in both English and Amharic. The questionnaire was divided into five sections covering (1) sociodemographic characteristics of the caregivers, (2) demographic and clinical characteristics of the children with epilepsy, 3–5) knowledge, attitudes and practices of the caregivers regarding epilepsy. To further ensure the accuracy of the data collection, two nurses working in the neurology clinic were recruited and provided with training by the investigators – a pediatrician and a child neurologist with extensive experience in managing CWE. This training focused on how to administer the questionnaire accurately and consistently.

### Outcome measures

#### Caregivers’ knowledge of epilepsy

Knowledge was evaluated using 7 knowledge-based questions:

1. What diagnosis does the child have? (enter term caregiver used in her/his own words)
2. Have you ever heard or read about the disease called ‘‘epilepsy’’?
3. Have you ever known anyone with epilepsy?
4. Have you ever witnessed a seizure?
5. Epilepsy is a form of.
6. What do you think is the cause of epilepsy?
7. Epilepsy is a contagious condition?

Knowledge was evaluated using the seven questions mentioned above. Caregivers were considered to have provided correct responses if they answered “Epilepsy” or “Seizure” to the question about the child’s diagnosis, indicated that they had heard or read about the disease, knew someone with epilepsy, had witnessed a seizure, recognized epilepsy as a form of brain disease, identified brain injury or birth injury as causes of epilepsy, and stated that epilepsy is never contagious.

Participants were categorized as having “good knowledge” if they answered ≥ 50% of questions correctly, while those scoring < 50% were classified as having “poor knowledge”.

### Caregivers’ attitudes towards epilepsy

Similarly, attitudes of participants were examined through 5 attitude-based questions:

1. A child with epilepsy can have a high level of intelligence.
2. A child with epilepsy should never attend school.
3. Would you allow your child to play with a child who has epilepsy?
4. Would you allow your son to marry a person with epilepsy?
5. Would you allow your daughter to marry a person with epilepsy?

Caregivers were considered to have provided correct responses if they indicated that a child with epilepsy can have a high level of intelligence, answered “false” to the statement that a child with epilepsy should never attend school, and expressed willingness to allow their child to play with a child who has epilepsy and to allow their son or daughter to marry a person with epilepsy.

Caregivers were categorized to have a “favorable attitude” if they answered favorably to ≥ 50% of attitude-based questions, whereas those scoring below 50% were deemed to have a “unfavorable attitude” towards epilepsy.

### Caregivers’ practices in managing treatment of epilepsy

The practices of participants were examined through four practice-based questions:

1. Has the child been treated with traditional treatments?
2. For seizures/epilepsy, is the child getting any other treatment besides the anti-seizure medication(s)?
3. At the onset of seizure where did you take the child for treatment/care?
4. If you had a friend or relative with epilepsy, what kind of treatment would you suggest?

### Statistical analysis

Data were entered into SPSS version 23 (IBM, Chicago, IL). Descriptive statistics were conducted to evaluate the frequency of the data; normally distributed measurement data were represented as mean ± standard deviation. To assess the correlation and association between KAP and demographic variables, the Chi-square (χ²) test was performed. The significance level was set at *p* < 0.05. In this study, convenience sampling was utilized to recruit caregivers of children with epilepsy (CWE) between May 1 and July 30, 2022, who met the predetermined inclusion and exclusion criteria. Convenience sampling was chosen due to its practicality and efficiency in accessing participants within the specified timeframe and criteria.

This study is reported following the STROBE guidelines [16].

## Results

### Participant characteristics

A total of 120 caregivers who cared for 120 children (one child per caregiver) were included in the study. Caregivers were aged between 18 and 61 years, with a mean age of 35.5 years and a standard deviation of 8.35 years. Of the caregivers, 82 (68.3%) were female. Regarding educational attainment, 32.5% of the participants have attained primary-level education while 45.8% attained secondary or post-secondary level of education. Full details of the caregivers’ characteristics are provided in Table 1.

**Table 1:** Characteristics of caregivers of children with epilepsy attending the outpatient clinic

Characteristics	*N*(%)	Mean (SD)
**Gender of the caregivers**		
Male	38(31.7)	
Female	82(68.3)	
**Age of caregivers in years**		35.51 (8.35)
18–35	69(57.5)	
36–45	38(31.7)	
≥ 46	13(10.8)	
**Caregivers’ relation to the child**		
Mother	78(65)	
Father	36 (30)	
Brother	2(1.7)	
Sister	1(0.8)	
Grand Mother	2(1.7)	
Aunt	1(0.8)	
**Child lives with**		
Mother	35(29.2)	
Father	6 (5)	
Both	75(62.5)	
Other	4(3.3)	
**Caregivers’ level of education**		
No education	26(21.7)	
Primary	39(32.5)	
Secondary	25(20.8)	
Tertiary or higher	30 (25)	
**Caregivers’ religion**		
Orthodox	82(68.3)	
Protestant	15(12.5)	
Muslim	23(19.2)	
**Caregivers’ marital status**		
Single	9(7.5)	
Married	84(70)	
Living together	13(10.8)	
Divorced/Widowed	14(11.7)	
**Caregivers Occupation Status**		
Farmer	8(6.7)	
Government Employed	19(15.8)	
Housewife	41(34.2)	
Private employed	36 (30)	
Others	16(13.3)	

The age of children with epilepsy being cared for ranged from 15 to 168 months (mean/SD 91.5/41.2 months). The majority of the children were male (79/120, 65.8%). Approximately 63% of the children lived with both parents. The full characteristics of the children can be found in Table 2.

**Table 2:** Characteristics of children with epilepsy attending the outpatient clinic

Characteristics	*N*(%)	Mean (SD)
**Gender**		
Male	79(65.8)	
Female	41(34.2)	
**Age (months)**		91.51(41.21)
**Age at onset of seizure (months)**		41.59(31.01)
**Duration of epilepsy in (months)**		51.34(35.35)
< 36	62(51.7)	
> 36	58(48.3)	
**School age children who attended school (> 5 years)**		
Yes	59(62.8)	
No	35(37.2)	
**Number of anti-seizure medications taken daily**		
One	81(67.5)	
Two	38(31.7)	
Three	1(0.8)	
**Type of anti-seizure medications**		
Carbamazepine	7(4.3%)	
Clonazepam	6(3.7%)	
Lamotrigine	3(1.9%)	
Phenobarbital	39(24.2%)	
Phenytoin	54(33.5%)	
Sodium Valproate	52(32.3%)	
**Comorbidities**		
Yes	34(28.3)	
No	86(71.7)	
**Type of Comorbidities**		
Attention-Deficit/Hyperactivity Disorder (ADHD)	3(8.8%)	
Autism Spectrum disorder (ASD)	10(29.4%)	
Cerebral palsy(CP)	20(58.8%)	
Intellectual disability(ID)	7(20.5%)	
**Family history of epilepsy**		
Yes	17(14.2)	
No	103(85.8)	

### Caregivers’ knowledge of epilepsy

In response to question 1, “What type of illness does the child have?”, 67.5% of caregivers used the term ‘epilepsy’ to describe the child’s condition. For question 2, “Have you ever heard or read about the disease called epilepsy?”, just over half of the caregivers had heard or read about epilepsy. For question 3, “Have you ever known anyone with epilepsy?”, half of the caregivers knew someone else with epilepsy. In response to question 4, “Have you ever witnessed a seizure?”, all caregivers reported having witnessed a seizure. The most common features of seizures witnessed by the caregivers were stiffness (85.8%), loss of consciousness (84.2%), tongue biting (59.2%), and confusion (40%).

When asked question 5, “What do you think epilepsy is a form of?”, approximately 36% of the respondents believed that epilepsy is a form of brain disease. For question 6, “What do you think is the cause of epilepsy?”, the most common cause reported by caregivers was birth injury (36.7%), followed by spirit possession (24.2%) and brain injury (23.3%). In response to question 7, “Is epilepsy a contagious condition?”, the majority of caregivers (83.3%) said epilepsy is never contagious, while 16.7% believed it is sometimes or always contagious. Only (13.3%) of caregivers provided correct responses to all the “knowledge” questions (Table 3).

**Table 3:** Knowledge assessment among caregivers of epileptic children at Yekatit 12 Hospital Medical College

Characteristics	Yes (%)	No (%)
Heard or read about epilepsy	62(51.7)	58(48.3)
Known anyone with epilepsy	61(50.8)	59(49.2)
**Cause of epilepsy**		
Brain injury	28(23.3)	92(76.7)
Curse from God	3(2.5)	117(97.5)
Runs in the family	4(3.3)	116(96.7)
Sprit possession	29(24.2)	91(75.8)
Birth injury	44(36.7)	76(63.3)
Blood disorder		120(100)
Excessive worry	3(2.5)	117(97.5)
Witchcraft	2(1.7)	118(98.3)
I don’t know	18 (15)	102(85)
**Epilepsy is a form of**		
Madness	3(2.5)	117(97.5)
Sprit or demon possession	32(26.7)	88(73.3)
Mental retardation	9(7.5)	111(92.5)
Brain disease	43(35.8)	77(64.2)
I don’t know	40(33.3)	80(66.7)
**Witnessed seizure**		
Loss of consciousness	101(84.2)	19(15.8)
Tongue biting	71(59.2)	49(40.8)
Stiffness	103(85.8)	17(14.2)
Loss of urine or stool	31(25.8)	89(74.2)
Confusion	48(40)	72(60)
Staring	26(21.7)	94(78.3)
**Epilepsy is a contagious condition**		
Always	6 (5)	
Sometimes	14(11.7)	
Never	100(83.3)	

### Associations between caregivers’ knowledge and demographic characteristics

Caregiver related factors associated with knowledge score include the sex of the caregivers (P value = 0.05), awareness of epilepsy through hearing or reading (P value < 0.001), knowing someone with epilepsy other than the index child (P value < 0.001), and level of knowledge (Table 4).

**Table 4:** Factors associated with knowledge related to epilepsy among parents/guardians of epileptic children at Yekatit 12 Hospital Medical College

Variables	Category	Level of Knowledge		Chi Square	*P* value
		Good(%)	Poor (%)		
Gender of the caregivers	Male	14(36.8)	24(63.2)	3.85	0.05*
	Female	46(56.1)	36(43.9)		
Age of the caregivers	18–35	34(49.3)	35(50.7)	0.812	0.66
	36–45	18(47.4)	20(52.6)		
	≥ 46	8(61.5)	5(38.5)		
Caregivers level of education	No education	9(34.6)	17(65.4)	4.89	0.18
	Primary	18(46.2)	21(53.8)		
	Secondary	15(60)	10(40)		
	Tertiary or higher	18(60)	12(40)		
Caregivers religion	Orthodox	36(43.9)	46(56.1)	4.80	0.09
	Protestant	8(53.3)	7(46.7)		
	Muslim	16(69.6)	7(30.4)		
Caregivers marital status	Single	2(22.2)	7(77.8)	4.99	0.173
	Married	44(52.4)	40(47.6)		
	Living together	5(38.5)	8(61.5)		
	Divorced/Separated	7(58.3)	5(41.7)		
Duration of epilepsy in month	< 36	33(53.2)	29(46.8)	0.534	0.465
	> 36	27(46.6)	31(53.4)		
Number of seizure medication taken daily	1	43(53.1)	38(46.9)	0.95	0.33
	≥ 2	17(43.6)	22(56.4)		
Comorbidities	Yes	17(50)	17(50)	0.001	1.00
	No	43(50)	43(50		
Family history of epilepsy	Yes	7(41.2)	10(58.8)	0.61	0.43
	No	53(51.5)	50(48.5)		
Heard or read about epilepsy	Yes	50(80.6)	12(19.4)	48.18	0.000*
	No	10(17.2)	48(82.8)		
Knew someone with epilepsy	Yes	50(82)	11 (18)	50.71	0.000*
	No	10(16.9)	49(83.1)		

* Significance at *P* < 0.05

### Caregivers’ attitudes towards epilepsy

Among caregivers, 56.7% thought that a child with epilepsy cannot attain high level of intelligence. The majority would not allow their child to play with a child who has epilepsy (76.7%) while 39.1% believed that a child with epilepsy should never attend school. The majority of caregivers would not allow their son 69.2% or daughter 68.3% to marry a person with epilepsy (Table 5).

**Table 5:** Caregivers’ attitudes towards children with epilepsy

Characteristics	Category	Frequency	Percent (%)
A child with epilepsy can have a high level of intelligence	Yes	52	43.3
	No	68	56.7
Would you allow your child to play with a child who has epilepsy	Yes	28	23.3
	No	92	76.7
A child with epilepsy should never attend school	Yes	47	39.1
	No	73	60.8
Would you allow your son to marry a person with epilepsy	Yes	37	30.8
	No	83	69.2
Would you allow your daughter to marry a person with epilepsy	Yes	38	31.7
	No	82	68.3

### Associations between caregivers’ attitudes and demographic characteristics

A higher percentage of caregivers with no family history of epilepsy exhibited unfavorable attitudes toward epilepsy compared to those with a family history (71.8% vs. 47.1%, *P* < 0.05) (Table 6).

**Table 6:** Factors associated with attitudes related to epilepsy among parents/guardians of epileptic children at Yekatit 12 Hospital Medical College

Variables	Category	Level of attitude		Chi Square	*P* value	
		Favorable	Unfavorable			
Gender of the caregivers	Male	11(28.9)	27(71.1)	0.19	0.66	
	Female	27(32.9)	55(67.1)			
Age of the caregivers	18–35	23(33.3)	46(66.7)	0.22	0.894	
	36–45	11(28.9)	27(71.1)			
	≥ 46	4(30.8)	9(69.2)			
Caregivers level of education	No education	12(46.2)	14(53.8)	4.80	0.187	
	Primary	8(20.5)	31(79.5)			
	Secondary	8 (32)	17(68)			
	Tertiary or higher	10(33.3)	20(66.7)			
Caregivers religion	Orthodox	27(32.9)	55(67.1)	0.41	0.815	
	Protestant	5(33.3)	10(66.7)			
	Muslim	6(26.1)	17(73.9)			
Caregivers marital status	Single	4(44.4)	5(55.6)	1.235	0.745	
	Married	26 (31)	58(69)			
	Living together	3(23.1)	10(76.9)			
	Divorced/Separated	3 (25)	9(75)			
Caregivers Occupation Status	Farmer	2 (25)	6(75)	0.876	0.928	
	Government Employed	6(31.6)	13(68.4)			
	Housewife	15(36.6)	26(63.4)			
	Private employed	10(27.8)	26(72.2)			
	Others	5(31.3)	11(68.8)			
Duration of epilepsy in month	< 36	19(30.6)	43(69.4)	0.062	0.804	
	> 36	19(32.8)	39(67.2)			
Number of seizure medication taken daily	1	26(32.1)	55(67.9)	0.022	0.883	
	≥ 2	12(30.8)	27(69.2)			
Comorbidities	Yes	14(41.2)	20(58.8)	1.983	0.159	
	No	24(27.9)	62(72.1)			
Family history of epilepsy	Yes	9(52.9)	8(47.1)	4.143	0.041*	
	No	29(28.2)	74(71.8)			
Heard or read about epilepsy	Yes	18 (29)	44(71)	0.411	0.521	
	No	20(34.5)	38(65.5)			
Knew someone with epilepsy	Yes	18(29.5)	43(70.5)	0.267	0.605	
	No	20(33.9)	39(66.1)			
Knowledge	Good	16(26.7)	44(73.3)	1.386	0.239	
	Poor	22(36.7)	38(63.3)			

* Significance at *P* < 0.05

### Caregivers’ practices in managing treatment of epilepsy

Of the caregivers, 29% accessed standard medical care in the treatment of their child’s epilepsy. However, 70.8% sought additional, non-standard treatment in addition to anti-seizure medications (e.g. holy places, traditional healers and home treatments).

When asked about where the child was taken for treatment/care at the onset of seizures, 68.3% caregivers reported taking the child to standard health facilities, e.g., local clinic or hospital, while 26.6% resorted to religious remedies such as “holy water”. Despite these discrepancies, 94.2% recommended seeking medical attention for individuals with epilepsy (Table 7).

**Table 7:** Caregivers’ practices in managing treatment of epilepsy

Characteristics		Category	Frequency	Percent (%)
Has the child been treated with traditional treatment		Yes	35	29.2
		No	85	70.8
For seizure is the child getting any other treatment besides anti-seizure medication		Yes	85	70.8
		No	35	29.2
At the onset of seizure where did you take the child for treatment		Health facilities	82	68.3
		Religious institutions	32	26.6
		Traditional healer	6	5.1
If you had a friend or relative with epilepsy what kind of treatment would you suggest	Take to holy water	Yes	29	24.2
		No	91	75.8
	See a doctor	Yes	113	94.2
		No	7	5.8
	See a traditional healer	Yes	9	7.5
		No	111	92.5

## Discussion

The aim of this study was to estimate knowledge, attitudes and practices among caregivers of children with epilepsy. The key findings of this study indicate that only (13.3%) of caregivers answered all the “knowledge” questions correctly (Table 3), most hold negative attitudes towards children with epilepsy (Table 5) and the large majority endorsed standard medical treatment-seeking practices for epilepsy, while a minority approved of concurrent traditional treatments.

### Caregiver’s knowledge

The present study is the first to assess the knowledge, attitudes, and practices specifically among the caregivers of CWE in Ethiopia. However, a significant gap in knowledge about epilepsy among the general population in Ethiopia has been observed in many previous studies, including in both urban and rural communities, as well as among preparatory school students, religious clerics, and teachers; these findings have been reinforced by meta-analyses [3, 9, 17–25].

In the present study, exactly half of respondents demonstrated a good level of epilepsy knowledge, which aligns with previous meta-analyses of the general Ethiopian population, which found good epilepsy knowledge to be 47.4% and 51.5%, respectively [24, 25]. This finding is somewhat unexpected, as one might anticipate that caregivers of CWE, especially those attending an outpatient neurology clinic, would have a relatively higher level of epilepsy knowledge due to their exposure to information about the disease.

Compared to caregivers in neighboring Sudan, where only 38.3% demonstrated good knowledge, a higher proportion of caregivers in this study had a good level of epilepsy knowledge [26]. This was observed despite similar distribution of educational achievement between the participants in both studies, with only 28.1% and 25% of studied caregivers attending university in Sudan and Ethiopia, respectively [26]. However, there were notable difference in the beliefs about epilepsy’s origins; a greater proportion (43.9%) of Sudanese caregivers endorsed supernatural origin, compared to only 24.2%, 2.5%, and 1.7% of Ethiopian caregivers attributing epilepsy to spirit possession, curse from God, or witchcraft, respectively [26]. These findings can also be compared to caregivers of CWE in northwestern Thailand, where only 14.5% reported a supernatural origin for epilepsy [27]. This discrepancy likely reflects strong cultural belief in the supernatural in Sudan and Ethiopia. Meanwhile, a smaller proportion of the presently studied caregivers believed that epilepsy is never contagious (83.3%), compared to those in Sudan (92.5%), and Thailand (95.2%) [26, 27].

In this study, no association was observed between caregivers’ level of educational attainment or occupational status and their epilepsy knowledge (Table 4). This contrasts with the study conducted by Legesse, et al., which found that lower educational attainment, occupation, and socioeconomic standing were association with worse epilepsy knowledge [19]. This finding may suggest that, despite the prominent poor epilepsy knowledge observed among caregivers, acting as a caregiver for a CWE results in overall increased exposure to clinical settings and information pertaining to epilepsy, which may be protective against the association between socioeconomic factors, such as wealth and education, and epilepsy knowledge seen in other studies.

### Caregiver’s attitudes

In the current study, family history of epilepsy was associated with more favorable attitudes towards CWE, while no association was observed between participants’ score on the knowledge questionnaire and their attitudes (Table 6). A similar discrepancy was observed in a population based study on epilepsy in Kuwait, where caregivers’ attitude were largely negative despite relatively higher knowledge scores [28, 29]. These observations suggest that caregivers’ negative attitudes persist even with adequate knowledge, contributing to the prominent stigmatization of epilepsy in Ethiopian society [12].

Interestingly, similar to the findings regarding knowledge, no association was seen between attitudes towards CWE and educational attainment or occupation of caregivers (Table 6). This underscores the potential protective role of being a caregiver for CWE against misconceptions related to socioeconomic variables, preventing negative attitudes as previously discussed.

Ethiopian children with epilepsy face stigma in many settings, including schools and churches. Negative attitudes and misconceptions are prevalent among important figures in their lives, such as classmates, teachers, religious clerics, and, as established in the present study, their own caregivers [17, 18, 30].Among the presently studied caregivers of CWE, more than half (56.7%) believe that children with epilepsy cannot be intelligent, and 39.1% say that children with epilepsy should not attend school (Table 5). Studies among preparatory school students have shown that negative attitudes are prevalent, such as the belief that epilepsy is a contagious disease or caused by an evil spirit, as well as an unwillingness to marry PWE, reflective of a lack of epilepsy knowledge contributing to negative attitudes [17]. Similar beliefs and attitudes were also prevalent among caregivers in the present study, though a greater proportion of caregivers reported birth injury or brain injury as the cause of epilepsy.

Negative attitudes regarding the intellectual potential of CWE observed in caregivers, and the association with supernatural phenomena observed among students have important implications, as CWE have been shown to have higher rates of school absenteeism related to seizures and must also overcome comorbid learning difficulties commonly associated with epilepsy [31]. Negative beliefs about the potential for academic achievement for CWE seen in the present study are likely influenced by the presence of comorbidities, as ASD and intellectual disability are among those most common. This highlights the importance of interventions such as after-school programs, tutoring and mentorship programs specifically aiming to support CWE with such comorbidities. In addition, children with epilepsy face persistent misconceptions, unfavorable attitudes, and stigmatization, which likely negatively impact their academic achievement [18, 31]. In the present study, 37.2% of the school-age children were not attending school, highlighting the importance of developing policies in Ethiopia that level the playing field by supporting and promoting the academic achievement of children with epilepsy. Furthermore, there is a need for information campaigns in settings such as schools and religious institutions that aim to reduce stigma by dispelling common misconceptions and negative attitudes toward people with epilepsy. Such initiatives could be a step forward in harm reduction for children with epilepsy in Ethiopia.

### Caregiver’s practices

The prevalence of poor knowledge and negative attitudes observed among the participants poses concerning implications for the safe practices pertaining to epilepsy, given previous studies conducted both within and outside of Ethiopia have shown an association between epilepsy medication non-adherence and factors such as caregivers’ knowledge and attitude toward epilepsy, perceived epilepsy-related stigma, self-stigma, low medication necessity belief, and negative medication belief [32–34]. In the present study, practices observed among caregivers were generally consistent with standard medical care, with 94.2% stating they would take a friend or relative with epilepsy to a doctor (Table 7). This is expected given the participants were sampled from caregivers of CWE currently receiving treatment at Yekatit 12 Hospital Pediatric Neurology Clinic and is likely indicative of strong medication belief. Nonetheless, a large majority (70.8%) stated that the child was receiving additional epilepsy treatment beyond anti-seizure medication, and nearly one-third of participants reported that at onset of seizures, they took their child to religious institutions (26.6%) or traditional healers (5.1%) (Table 7). A similar proportion also indicated that if a friend or relative developed epilepsy, they would suggest seeing a traditional healer (7.5%) or taking holy water as treatment (24.2%), though these were not mutually exclusive with seeing a doctor as mentioned previously (Table 7). These findings indicate that despite strong medication belief, the strong cultural belief in the supernatural, as previously described, is profoundly entrenched in Ethiopian society and results in persistence of the pursuit of non-medical treatments for CWE. This is significant and suggests clinical practice should focus on finding ways to ensure alternative treatments are pursued safely and without disrupting medical treatment, rather than attempting to alter caregivers’ deeply held cultural beliefs.

### Limitations

This study population was limited to those caregivers who accessed medical treatment for their child at the outpatient clinic, which will impact on generalizability of the results. Furthermore, since the study only included caregivers already seeking standard medical care for their children, the sample is not representative in terms of providing information on knowledge, attitudes and practices of those who either do not have access to medical care or choose not to seek standard medical care. As such, this study likely overestimates the prevalence of good knowledge, favorable attitudes, and safe practices among caregivers of CWE. Thus, it is particularly unsurprising that practices reported in the present study were generally non-life endangering, since caregivers had already elected to seek medical treatment in order to be included in this study. In addition, a sample size calculation was not performed, and it is not clear if the reported associations were sufficiently powered.

## Conclusions

The study provides up-to-date insights into knowledge, attitudes and practices among caregivers of children with epilepsy, and highlights the need to address poor knowledge, negative attitudes and specific misconceptions held by caregivers of CWE. These insights should guide the development of educational interventions designed specifically for caregivers, such as workshops, educational applications and online resources which address these specific gaps in such a way that they are accessible to a broad range of caregivers, including those living outside of urban environments. The present findings may also enhance clinicians’ understanding of caregiver perspectives as well as the social and cultural factors that influence epilepsy-related knowledge and attitudes. This should in turn improve communication between caregivers and physicians and enhance caregiver engagement, fostering an improved, collaborative, and therapeutic relationship between clinician, patient, and caregiver. Beyond this, understanding the specific gaps in knowledge will enable focused interventions at the community level, and should guide policy changes, particularly increased resource allocation for epilepsy care in the pediatric population. Further studies should explore the root causes of the prevailing negative attitude toward epilepsy with qualitative studies such as focus group discussions.

### Electronic supplementary material

Below is the link to the electronic supplementary material.

Supplementary Material 1

## Data Availability

No datasets were generated or analysed during the current study.

## Abbreviations

CWE: Children with epilepsy
IRB: Institutional Review Board
KAP: Knowledge, attitude and practice
PI: Principal investigator
PWE: People with epilepsy
SPSS: Statistical Package for Social Sciences
