# Supplementary material for: Knowledge, attitudes, and practices of caregivers with children diagnosed with epilepsy attending a pediatric outpatient clinic: a descriptive, cross-sectional, questionnaire-based study in Addis Ababa, Ethiopia
Source: BMC Neurol. 2024 Jul 22;24:252. doi: 10.1186/s12883-024-03766-1 (PMC11265119; doi:10.1186/s12883-024-03766-1)
Supplement: Supplementary file 1 — Supplementary Material 1 [file 12883_2024_3766_MOESM1_ESM.docx]

**Knowledge, attitudes, and practices of caregivers with children diagnosed with epilepsy attending a pediatric outpatient clinic: a descriptive, cross-sectional, questionnaire-based study in Addis Ababa, Ethiopia.**

1. **From caregivers: Caregiver related variables**

| **Demography** | |  |
| --- | --- | --- |
| 1 | Age of caregiver |  |
| 2 | Caregiver’s gender | Male / Female |
| 3 | Caregiver’s relation to child |  |
| 4 | Who does the child live with? | Mother |
|  |  | Father |
|  |  | Both |
|  |  | Other (specify) |
| 5 | Do you live with the child ? | Yes |
|  |  | No , if no how often do you see the child |
| 6 | Caregivers level of education | No education |
|  |  | Primary |
|  |  | Secondary |
|  |  | Tertiary or higher |
| 7 | Caregivers occupation |  |
| 8 | Caregiver’s religion | Orthodox |
|  |  | Catholic |
|  |  | Protestant |
|  |  | Muslim |
|  |  | Other (Specify) |
| 9 | Caregiver’s marital status | Never married |
|  |  | Married |
|  |  | Living together |
|  |  | Divorced/separated |
|  |  | Widowed |

1. **Demographic and clinical characteristics of the children with epilepsy**

| 1 | Date of birth / Age of the child |  |
| --- | --- | --- |
| 2 | Gender | Male / Female |
| 3 | Age at onset of seizures |  |
| 4 | Duration of epilepsy in months |  |
| 5 | Is the child in school? |  |
| 6 | Number of ASM taken daily |  |
| 7 | List of anti-seizure medications (ASM) |  |
| 8 | Known comorbidities | Yes , if yes specify |
|  |  | No |
| 9 | Family history of epilepsy | Yes |
|  |  | No |

1. **Knowledge of the caregivers regarding epilepsy**

| 1 | What diagnosis does the child have? (enter term caregiver used in her/his own words) |  |
| --- | --- | --- |
| 2 | Have you ever heard or read about the disease called ‘‘epilepsy’’? |  |
| 3 | Have you ever known anyone with epilepsy? |  |
| 4 | Have you ever witnessed a seizure? (if yes, list them) |  |
| 5 | ‘‘Epilepsy is a form of’’: |  |
| 6 | What do you think is the cause of epilepsy? |  |
| 7 | Epilepsy is a contagious condition. |  |

1. **Attitude of the caregivers regarding epilepsy**

| 1 | ‘‘A child with epilepsy can have a high level of intelligence.’’ | True |
| --- | --- | --- |
|  |  | False |
| 2 | ‘‘A child with epilepsy should never attend school.’’ | True |
|  |  | False |
| 3 | Would you allow your child to play with a child who has epilepsy? | Yes |
|  |  | No |
| 4 | Would you allow your son to marry a person with epilepsy? | Yes |
|  |  | No |
| 5 | Would you allow your daughter to marry a person with epilepsy? | Yes |
|  |  | No |

1. **Caregivers practice in managing a child with epilepsy**

| 1 | Has the child been treated with traditional treatments? | Yes |
| --- | --- | --- |
|  |  | No |
| 2 | For seizures/epilepsy, is the child getting any other treatment besides the anti-seizure medication (s)? |  |
| 3 | At the onset of seizure where did you take the child for treatment/care? |  |
| 4 | If you had a friend or relative with epilepsy, what kind of treatment would you suggest? |  |

Thank you for participating in this survey. We welcome any comments you have regarding this survey. Please place them below.
